# Functional Characterization of the Infection-Inducible Peptide Edin in *Drosophila melanogaster*

**DOI:** 10.1371/journal.pone.0037153

**Published:** 2012-05-14

**Authors:** Leena-Maija Vanha-aho, Anni Kleino, Meri Kaustio, Johanna Ulvila, Bettina Wilke, Dan Hultmark, Susanna Valanne, Mika Rämet

**Affiliations:** 1 BioMediTech and Institute of Biomedical Technology, University of Tampere, Tampere, Finland; 2 Department of Pediatrics, and Biocenter Oulu, University of Oulu, Oulu, Finland; 3 Department of Molecular Biology, Umeå University, Umeå, Sweden; 4 Department of Pediatrics, Tampere University Hospital, Tampere, Tampere, Finland; French National Centre for Scientific Research – Université Aix-Marseille, France

## Abstract

Drosophila is a well-established model organism for studying innate immunity because of its high resistance against microbial infections and lack of adaptive immunity. In addition, the immune signaling cascades found in Drosophila are evolutionarily conserved. Upon infection, activation of the immune signaling pathways, Toll and Imd, leads to the expression of multiple immune response genes, such as the antimicrobial peptides (AMPs). Previously, we identified an uncharacterized gene edin among the genes, which were strongly induced upon stimulation with Escherichia coli in Drosophila S2 cells. Edin has been associated with resistance against Listeria monocytogenes, but its role in Drosophila immunity remains elusive. In this study, we examined the role of Edin in the immune response of Drosophila both in vitro and in vivo. We report that edin expression is dependent on the Imd-pathway NF-κB transcription factor Relish and that it is expressed upon infection both in vitro and in vivo. Edin encodes a pro-protein, which is further processed in S2 cells. In our experiments, Edin did not bind microbes, nor did it possess antimicrobial activity to tested microbial strains in vitro or in vivo. Furthermore, edin RNAi did not significantly affect the expression of AMPs in vitro or in vivo. However, edin RNAi flies showed modestly impaired resistance to E. faecalis infection. We conclude that Edin has no potent antimicrobial properties but it appears to be important for E. faecalis infection via an uncharacterized mechanism. Further studies are still required to elucidate the exact role of Edin in the Drosophila immune response.

## Introduction

Innate immunity is the first line of defense in all multicellular organisms. During the last few decades, the fruit fly *Drosophila melanogaster* has proven to be well suited for studying innate immune responses. In contrast to vertebrates, *Drosophila* only has an innate immune system, which is highly sophisticated and in part conserved among higher organisms [1]. In *Drosophila*, effective innate immune responses are based on the ability of several pattern-recognition receptors to recognize and bind common microbial surface structures. One main outcome of this initial microbial recognition is the activation of NF-κB immune signaling pathways, which leads to the production of several potent antimicrobial peptides (AMPs).

In *Drosophila*, the production of AMPs is mainly regulated by two NF-κB signaling pathways: the Imd (immune deficiency) pathway [2] reviewed in [3] and the Toll pathway [4] reviewed in [5]. Both of these pathways are highly conserved from fly to man. The Imd pathway is activated by diaminopimelic acid-type peptidoglycan (DAP) [6], present in most or all Gram-negative bacteria, but also in some Gram-positive bacteria like *Listeria monocytogenes*. The Toll pathway is activated mainly by the lysine-type peptidoglycan present in many other Gram-positive bacteria [7], reviewed in [5]. Both of these signaling pathways can also be induced by different fungi [8], [9]. Activation of the Imd and Toll signaling pathways upon microbial infection ultimately causes the nuclear translocation of the NF-κB transcription factors, Relish or Dif/Dorsal respectively, leading to the expression of dozens of NF-κB responsive genes [10], [11], [12], [13], [14]. The molecular function of many of these genes still remains unknown.

Earlier, we identified a gene, *CG32185*, to be highly induced in S2 cells in response to heat-killed *Escherichia coli* [14]. Later, Gordon et al. called the gene *edin* and found it to be associated with *Listeria monocytogenes* resistance [15]. In addition, it has been shown that Edin is secreted into the hemolymph in *Drosophila* third instar larvae upon infection [16]. Because the molecular function of Edin and the signaling pathways involved are still mainly unknown, in our current study we set out to examine the role of Edin in the *Drosophila* immune response both *in vitro* and *in vivo*.

## Results

### *Edin* expression is Relish-dependent *in vitro* and *in vivo* upon Gram-negative bacterial infection

When *Drosophila* encounters microbes, several signaling pathways are activated leading to transcriptional modifications. This response varies depending on the microbe and the site of infection. During a systemic infection, the expression of dozens of genes is induced [11], [12] leading to very effective defense responses. Upon infection, most of the highly induced genes are known to be *AMP* genes, *DIM*s (Drosophila immune-induced molecules) or genes related to signal regulation. Nevertheless, the molecular function of several of the induced genes is yet to be characterized. Previously, we studied which genes are induced in response to heat-killed *Escherichia coli* in *Drosophila* macrophage-like S2 cells [14]. Table I represents the oligonucleotide microarray data of the most strongly induced genes (data collected from [14]). The eight most strongly induced genes encode five known AMPs, one peptidoglycan recognition protein (*PGRP-LB*), a negative regulator of the Imd pathway (*pirk*) [17] and *edin* (CG32185). According to the microarray results, the expression of *edin* is strongly induced within hours after the bacterial challenge and the induction pattern of *edin* resembles that of known antimicrobial peptides (Table I).

**Table 1:** Induction of *Drosophila* antimicrobial peptide genes and *edin* in *E. coli* -challenged S2 cells (data collected from [14]).

Gene	#CG	0 h	0.5 h	1 h	4 h	24 h	*Relish* RNAi 4 h
*Attacin B*	CG18372	1±0.1	1.5	6.0	60.6±15.1	87.2±87.2	0.1±0.0
*Diptericin B*	CG10794	1±0.0	2.3	3.6	52.4±4.3	78.1±1.8	0.2±0.1
*Attacin D*	CG7629	1±0.0	1.1	2.6	47.5±6.3	92.5±2.1	0.1±0.1
*Metchnikowin*	CG8175	1±0.0	1.7	6.5	41.3±15.7	52.2±2.0	0.4±0.1
*Edin*	CG32185	1±0.1	0.9	3.5	29.8±6.4	48.5±1.1	0.0±0.0
*Pirk*	CG15678	1±0.2	2.0	15.5	15.1±0.6	5.4±0.0	0.4±0.1
*PGRP-LB*	CG14704	1±0.0	1.2	2.0	8.5±2.0	20.7±0.3	0.7±0.2
*Cecropin B*	CG1878	1±0.0	1.2	3.2	7.4±0.9	4.0±0.3	0.6±0.0

In S2 cells, the response to *E. coli* is known to be predominantly mediated via the Imd pathway [13]. To verify whether the induction of *edin* is dependent on the Imd pathway, we silenced the Imd pathway by knocking down the transcription factor Relish by RNAi. The induction of *edin* was completely abolished in *Relish* dsRNA treated S2 cells at the 4 h time point (Table I) indicating that *edin* expression is regulated via the Imd pathway in S2 cells after induction with heat-killed *E. coli*.

**Figure 1:**
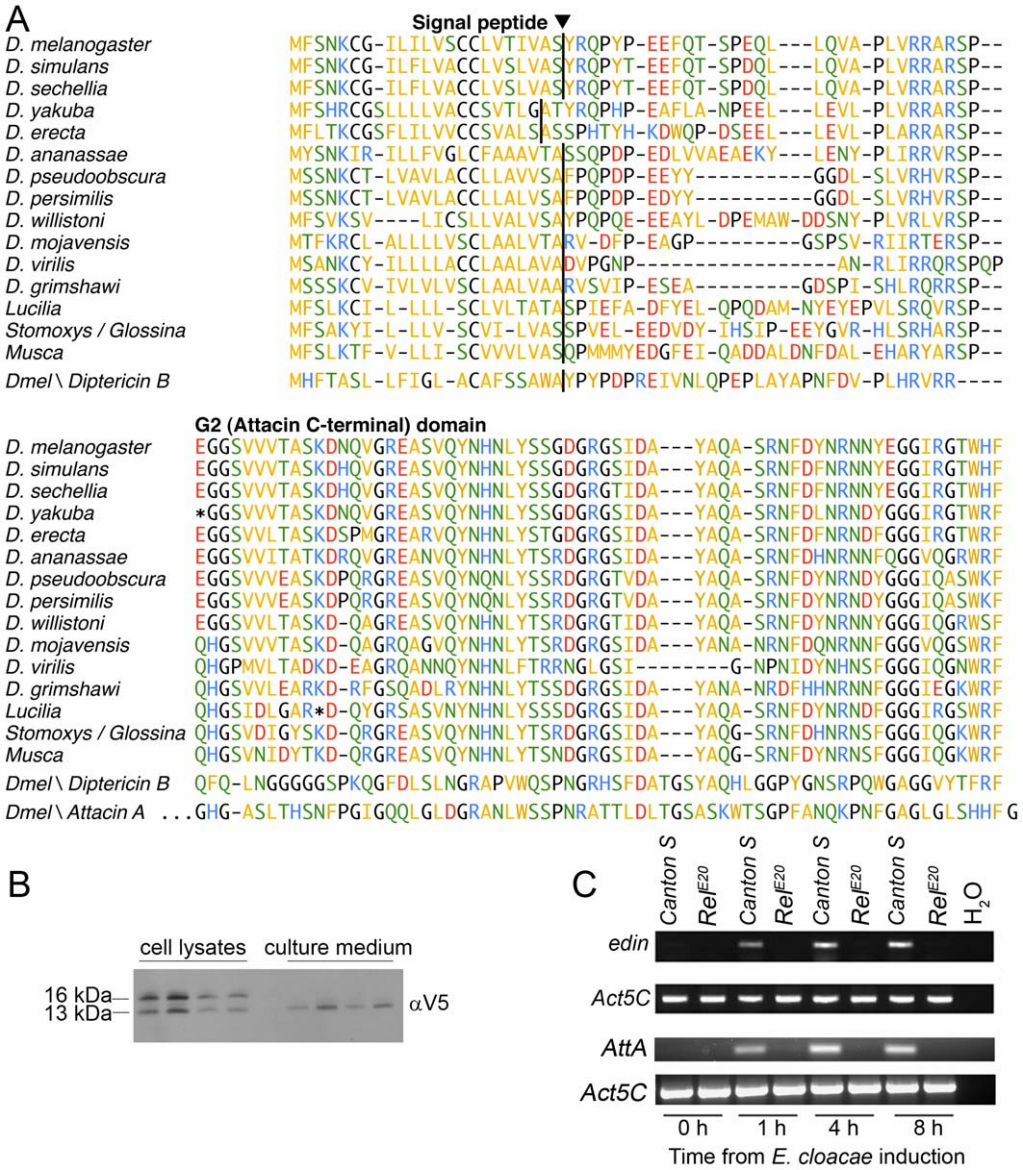
Edin is a Relish-dependently synthesized peptide, which is secreted from S2 cells. (A–B) Edin contains a signal sequence and is secreted from S2 cells. (A) Edin sequences are aligned from 12 *Drosophila* species and three other dipterans. Diptericin B and Attacin A from *D. melanogaster* are also included in the alignment. The predicted signal peptidase cleavage sites [31] are marked. The sequences from the 12 *Drosophila* species are all from Clark et al. 2007 [32], except the *D. mojavensis* sequence which is derived from an EST sequence (EB600147). Modified gene models without introns were used for *D. yakuba* and *D. willistoni*. The *Lucilia sericata* sequence is derived from a single EST (FG360503). Three *Stomoxys calcitrans* ESTs (DN952426, DN952940, EZ048833) and one *Glossina morsitans* EST (AF368915) appear to contain overlapping sequence from the same gene. The *Musca domestica* sequence is an isoform represented by one EST (ES608713). (B) The signal sequence of Edin is cleaved before the peptide is secreted to the cell culture medium. S2 cells were transfected with a pMT-*edin*-V5 construct and the cell culture medium and cell lysates were analyzed with western blotting. Both full-length and cleaved forms were observed in the lysates while only the cleaved form was present in the medium. The V5 tag is located at the C-terminus of Edin. The blot represents 4 independent samples from which both cell lysates and culture medium were analyzed. (C) *Edin* is induced upon *Enterobacter cloacae* infection in *Canton S* flies but not in *Rel^E20^* flies. Canton S flies and *Rel^E20^*-mutant flies were pricked with *E. cloacae* and total RNAs were extracted at the indicated time points. RT-PCR was performed and samples were electrophoresed on an agarose gel. *Actin5C* was used as a loading control and *Attacin A* as a positive control.

The *edin* gene encodes a short peptide of 115 amino acids including an N-terminal signal sequence (amino acids 1–22) (Figure 1A). The predicted signal peptidase cleavage site is supported by proteomic data from Verleyen et al. [16], who identified the predicted amino terminal of the mature protein in peptide fragments from hemolymph. Likely orthologs of the *edin* gene can be found in other brachyrecan flies, including all sequenced *Drosophila* species, but not in other insects (Figure 1A). For *Musca domestica*, three isoforms are represented in the EST databases (not shown). A tendency for pseudogenisation of the *edin* genes can be noted, as stop codons are present in the *D. yakuba* and *D. mojavensis* homologs. For the latter, an apparently functional allele is represented by an EST sequence (Figure 1A). A stop codon interrupts the open reading frame in the EST from *Lucilia sericata*, but this could be a sequencing error.

Iterated PSI-BLAST searches indicate that Edin is related to the Attacin/Diptericin superfamily of glycine-rich antibacterial peptides. The best hits were to *Drosophila virilis* Diptericin B (E = 8e-20) and *Hyalophora cecropia* Attacin E (E = 2e-18). Figure 1A shows an alignment to Diptericin B and the C-terminal (G2) domain of Attacin A from *D. melanogaster*.

Since Edin has a predicted signal sequence, we next examined if Edin is actually secreted from cells. To test this, we cloned *edin* cDNA into the heavy metal-inducible expression vector pMT/V5, transfected S2 cells with the construct and analyzed the presence of the protein both in the cell culture medium and cell extracts by western blotting using an anti-V5 antibody. In the S2 cells, both shorter and longer forms of Edin were detected, corresponding to V5-tagged peptides with and without the signal sequence, respectively. In the cell culture medium, only the shorter, C-terminal form, without the signal sequence could be observed (Figure 1B). This result suggests that Edin has a functional signal sequence, which is cleaved before the peptide is secreted. These results are in line with the report of Verleyen and coworkers [16], who detected amino-terminal fragments of Edin with mass spectrometry in the hemolymph of *Drosophila* larvae infected with a mixture of Gram-negative and Gram-positive bacteria.

Since the expression of *edin* is Relish-dependent *in vitro*, we next investigated whether *edin* is also induced upon microbial challenge *in vivo*. We infected wild-type *Canton S* and *Relish* null mutant adult flies (*Rel^E20^*) with the Gram-negative bacteria *Enterobacter cloacae*. Total RNAs were extracted and the transcript levels of *edin* were determined with RT-PCR and agarose gel electrophoresis. As shown in Figure 1C, *edin* is induced in *Canton S* but not in *Rel^E20^* mutant flies. *Attacin A* was used as a positive control and showed a similar expression pattern to *edin* (Figure 1C). These results together with the previously published microarray data indicate that *edin* expression is strongly and rapidly induced upon a Gram-negative bacterial infection in a Relish-dependent manner both *in vitro* and *in vivo*. These results together propose that Edin has a function related to microbial resistance. Thus, we next subjected Edin to further functional characterization both *in vitro* and *in vivo*.

### Edin has no significant effect on bacterial binding

**Figure 2:**
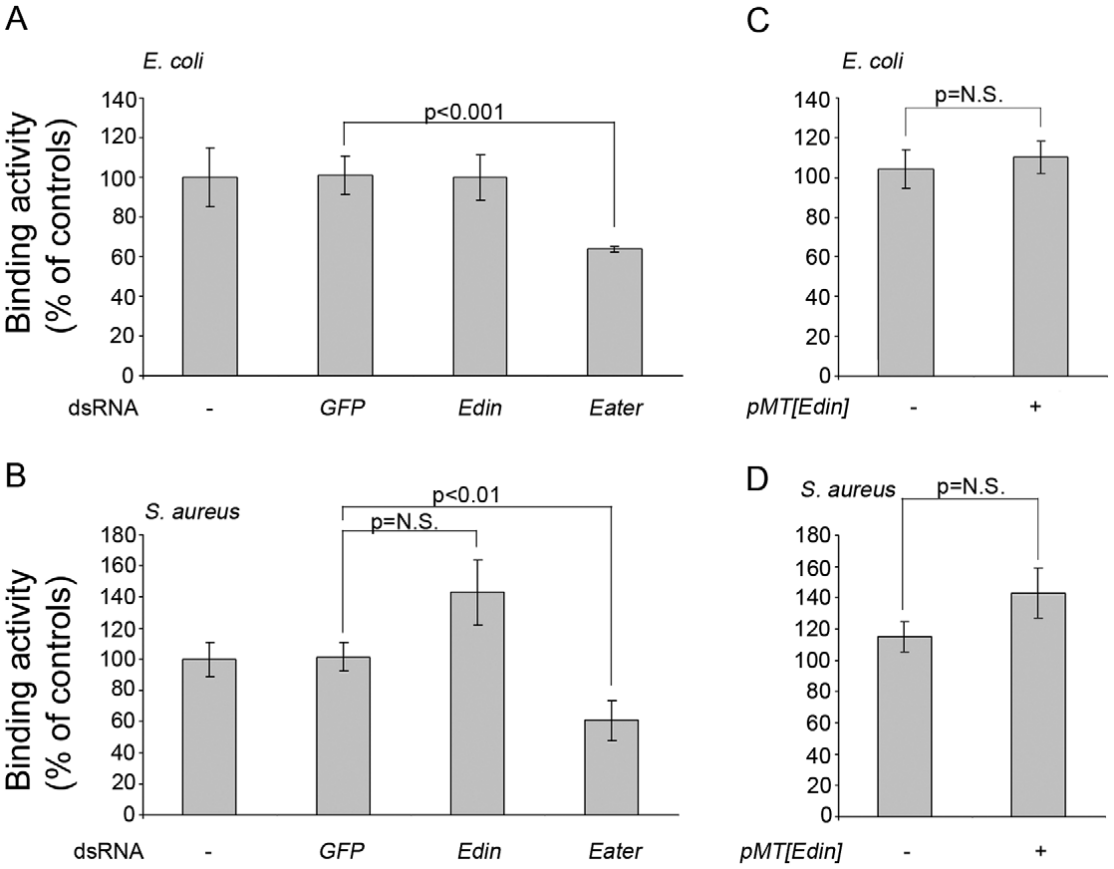
Edin does not affect the ability of S2 cells to bind microbes. (A–B) The effect of *edin* RNAi on the binding of *E. coli* and *S. aureus* in *Drosophila* S2 cells. *Drosophila* S2 cells were soaked for three days in dsRNAs and thereafter exposed to bacteria at +4°C. *GFP* dsRNA was used as a negative and *eater* dsRNA as a positive control. (C–D) The effect of *edin* overexpression on the binding of *E. coli* and *S. aureus.* S2 cells were transiently transfected with a pMT construct expressing *edin* and endogenous *edin* expression was knocked down with dsRNA treatments. The ability of S2 cells to bind heat-killed *E. coli* (A, C) or *S. aureus* (B, D) was measured using flow cytometry.

The phagocytosis of invading microbes is an essential component of *Drosophila* immunity [18], [19]. To this end we tested whether Edin has a role in bacterial binding or opsonization. Plasmatocyte-like S2 cells that are capable of binding and phagocytosing microbes [20] were treated with *edin* dsRNA and the ability of the cells to bind heat-killed, fluorescently labeled *E. coli* and *Staphylococcus aureus* was analyzed with flow cytometry. As a positive control, we used a dsRNA treatment targeting *eater*, which codes for an important phagocytic receptor for bacteria both in S2 cells and in *Drosophila in vivo* [18], [19], [21]. *GFP* dsRNA was used as a negative control. *Edin* RNAi did not affect the ability of S2 cells to bind *E. coli* (Figure 2A). Likewise, *edin* dsRNA treatments did not compromise the ability of S2 cells to bind *S. aureus* (Figure 2B) but rather seemed to modestly enhance the binding activity of S2 cells.

To test the effect of *edin* overexpression on bacterial binding, S2 cells were first transiently transfected with a pMT[*edin*]V5 construct. An empty pMT/V5 plasmid was transfected as a control. 24 h after transfection, CuSO_4_ was added to the cell culture medium to induce the expression of the construct. Two days later, the medium was collected and transferred to other S2 cells which were pre-treated with *edin* dsRNA to block endogenous *edin* expression. Thereafter, FITC-labeled, heat-killed *E. coli* or *S. aureus* were added and the amount of cell-associated bacteria was monitored using flow cytometry. In line with the results of *edin* RNAi experiments, *edin* overexpression had no effect on the binding of *E. coli* (Fig. 2C) or *S. aureus* (Fig. 2D). The presence of Edin in the cell-culture medium was confirmed by western blotting using an anti-V5 antibody (data not shown).

**Figure 3:**
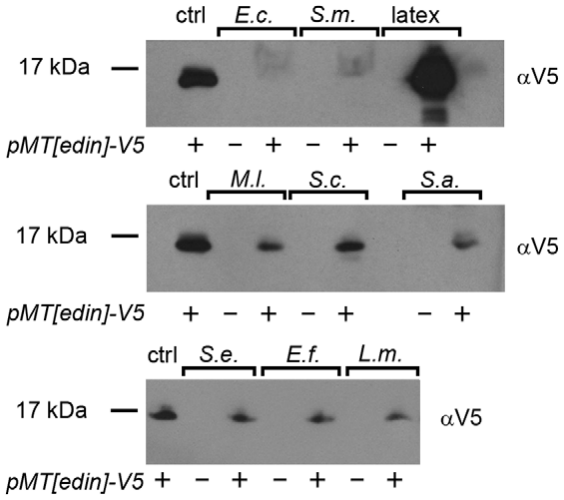
The effect of Edin on microbial binding. 500 µl of Edin-V5 containing medium were incubated with 1 ml of a bacterial suspension of live *E. coli* (E.c.), *Serratia marcescens* (S.m), *Staphylococcus epidermidis* (S.e.), *Enterococcus faecalis* (E.f.), *Listeria monocytogenes* (L.m.), *Micrococcus luteus* (M.l.), *Saccharomyces cerevisiae* (S.c.) or *S. aureus* (S.a) for 1 h with mild agitation at +4°C. Latex beads treated with BSA were used as a control. The samples were then centrifuged and the pellet was washed. Edin bound to microbes was detached by adding 20 µl of SDS-PAGE loading buffer, boiled for 10 minutes, electrophoresed on SDS-PAGE and detected using a V5 antibody. The first lane of each blot is a control sample containing 20 µl of Edin-V5 medium. The following lanes contain 30 µl of the medium incubated with the indicated microbe.

To investigate in a more direct way if Edin binds microbes, we incubated Edin-containing cell culture medium with live *E. coli*, *Serratia marcescens*, *Staphylococcus epidermidis*, *Enterococcus faecalis*, *Listeria monocytogenes*, *Micrococcus luteus*, *Saccharomyces cerevisiae* and *S. aureus*. Latex beads (carboxylated polystyrene), which are expected to bind all kinds of proteins to some extent, were used as a positive control. The microbial suspensions were incubated with 500 µl of Edin-containing medium at +4°C after which the microbes were pelleted and washed with PBS. Finally, the pellets were suspended and boiled in an SDS-PAGE sample buffer to detach bound Edin from the microbes before electrophoresis. Next, the proteins were transferred onto nitrocellulose membranes and Edin was detected using an anti-V5 antibody. As a reference, 20 µl of Edin-containing medium was loaded into the first lane. Therefore, if Edin attached efficiently to the indicated microbe, much more Edin should be detected in the samples (500 µl Edin-containing medium used) compared to the reference lane (20 µl Edin-containing medium). As shown in Figure 3 (the rightmost lanes), carboxylated latex beads, i.e. the positive control, bound Edin. In contrast, virtually no Edin was bound to the tested Gram-negative bacteria, *E. coli* and *S. marcescens*. Furthermore, only a faint signal was detected with the Gram-positive bacteria *S. epidermidis*, *E. faecalis*, *L. monocytogenes*, *M. luteus* and *S. aureus*, and with the baker's yeast *S. cerevisiae* as compared to the reference lane (ctrl in Figure 3). Based on these results, we conclude that Edin does not strongly bind any of the tested microbes.

### The effect of Edin on immune signaling

**Figure 4:**
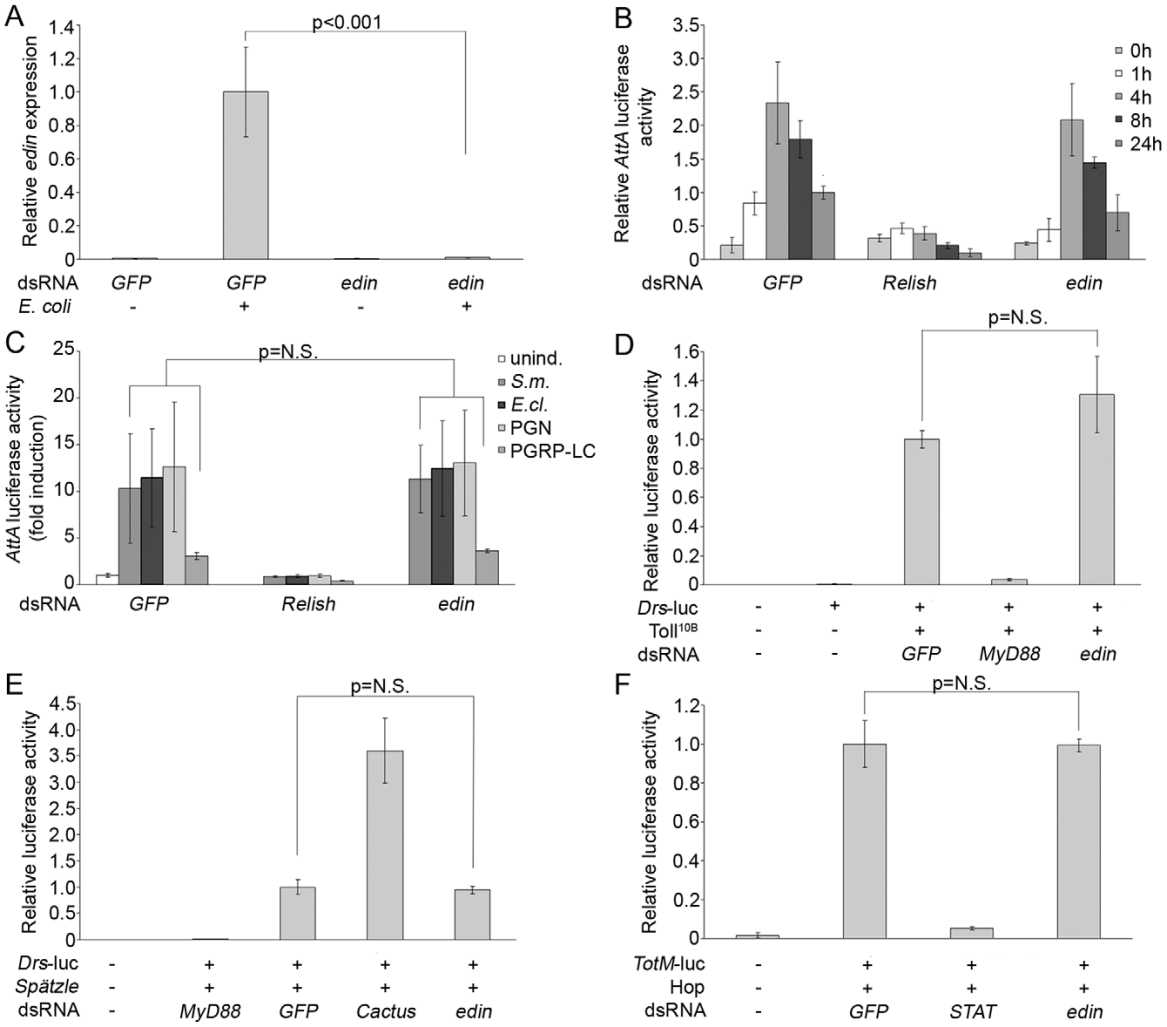
Effect of *edin* RNAi on *Drosophila* immune signaling in vitro. A) *Edin* RNAi is effective in S2 cells. S2 cells were treated with *GFP* and *edin* dsRNA and the cells were induced by adding heat-killed *E. coli*. Relative expression levels of *edin* were analyzed from total RNAs with qRT-PCR. n = 4 for each sample. (B) *Edin* expression is not required for the Imd pathway signaling *in vitro*. S2 cells were transfected with an *Attacin A-*luciferase reporter together with *GFP* (negative control), *Relish* (positive control) and *edin* dsRNAs. The Imd pathway was activated by adding heat-killed *E. coli* to the cell culture medium and samples were collected at indicated time points. *Edin* RNAi causes a 30% decrease in the Imd pathway activity at the 24 h time point. The data for the 0 h and 24 h time points are pooled from 5 indepent experiments (n = 17 per sample). For 1 h, 4 h and 8 h time points n = 4 per sample. (C) *Edin* RNAi does not decrease the Imd pathway activity when the pathway is induced with *S. marcescens*, *E. cloacae*, peptidoglycan or PGRP-LC. S2 cells were transfected with an *AttA*-luciferase reporter and *edin* dsRNA and the Imd pathway was activated with *S. marcescens* (*S.m.*), *E. cloacae* (*E.cl.*), peptidoglycan (PGN) or a *pMT[PGRP-LC]* construct. CuSO_4_ was used to induce the expression of *PGRP-LC*. *GFP* and *Relish* dsRNAs were used as negative and positive controls, respectively. Unind. = no induction. The data for *S.m.*, *E.cl.* and PGN are pooled from 3 independent experiments (n = 12 per sample). For PGRP-LC, n = 3 per sample. (D) *Edin* RNAi does not affect the Toll pathway activity. S2 cells were transfected with a *Drosomycin*-luciferase reporter together with *GFP*, *edin* and *MyD88* (positive control) dsRNAs. A constitutively active form of the Toll receptor, Toll^10B^, was used to activate the pathway. The data are pooled from 3 independent experiments, n = 10 for each sample. (E) *Edin* has no effect on the Spätzle-induced Toll-pathway activity. S2 cells were transfected with a *Drosomycin*-luciferase reporter together with *GFP*, *edin*, *MyD88* (control) and *Cactus* (control) dsRNAs. The Toll pathway was activated with the cleaved, active Spätzle ligand (*Spz^C106^*). n = 4 for each sample. (F) *Edin* RNAi has no effect on the JAK/STAT pathway. S2 cells were transfected with a *Turandot M*-reporter and *GFP*, *STAT* (positive control) and *edin* dsRNAs. The JAK/STAT pathway was activated by overexpressing *Hop^Tum-l^*. n = 4 for each sample.

Next, we investigated whether Edin is involved in modulating the activity of *Drosophila* innate immune signaling cascades. S2 cells were transfected with luciferase-reporter constructs together with *edin* dsRNA as well as with negative and positive control dsRNAs, and the luciferase activities of the cell lysates were analyzed. Transfection efficacy and cell viability were assessed with an *Actin 5C*-β-galactosidase reporter. *GFP* dsRNA was used as a negative control in all assays. First, we tested the effectiveness of *edin* RNAi *in vitro* by treating S2 cells with *GFP* or *edin* dsRNAs, and analyzing the relative expression levels of *edin*. As shown in Figure 4A, *edin* RNAi abolishes the endogenous *edin* expression.

In order to analyze the Imd pathway activity, an *Attacin A*-luciferase reporter and *Relish* dsRNA as a positive control were used and the pathway was activated by adding heat-killed *E. coli* to the cell culture medium. The samples were collected 0 h (no induction), 1 h, 4 h, 8 h and 24 h after *E. coli* induction. As expected, *Relish* RNAi strongly decreases the Imd-pathway activity at all time points (Figure 4B). On the contrary, *edin* RNAi had minor or no effect in this setting, although at the 24 h time point there was a trend for reduced *Attacin A* promoter driven luciferace activity (Figure 4B). Because *edin* RNAi appeared to have a minor effect on the Imd pathway activity when induced with heat-killed *E. coli* at the 24 h time point, we next investigated the effect of the *edin* dsRNA with other pathway elicitors. To this end, heat-killed *S. marcescens*, heat-killed *E. cloacae*, peptidoglycan and overexpression of the cytoplasmic tail of the PGRP-LC receptor were used. As shown in Figure 4C, *edin* RNAi had no effect on the *AttA*-luciferase activity in this experimental setting. These results indicate that Edin does not have an important role in the regulation of the Imd pathway activity in S2 cells.

To investigate the role of Edin in the Toll pathway signaling, we used a *Drosomycin*-luciferase reporter, and *MyD88* dsRNA as a positive control, and activated the pathway by transfecting the cells with a constitutively active form of the Toll receptor, *Toll^10B^* (Figure 4D) or with the cleaved, active Spätzle ligand (Figure 4E). For the JAK/STAT signaling pathway, we used *TurandotM*-luciferase reporter and *STAT* dsRNA as a positive control (Figure 4F). The pathway was activated by overexpressing *hopscotch^Tum-l^*, the active form of *Drosophila* Jak. *Edin RNAi* did not significantly affect the signaling via the Toll pathway (Figure 4D–E), or the JAK/STAT pathway (Figure 4F). These results indicate that Edin has no central role in regulating immune signaling *in vitro*.

**Figure 5:**
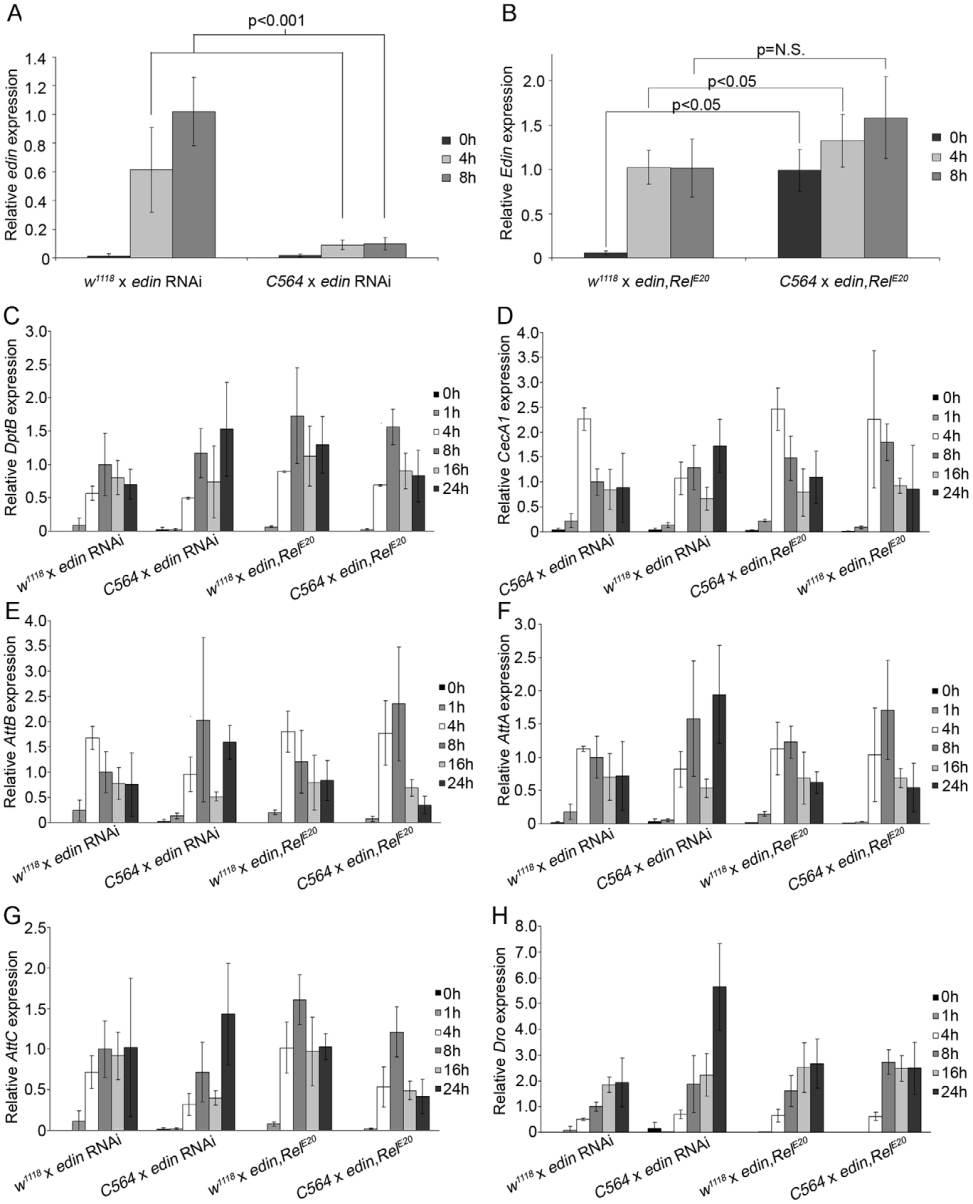
The effect of Edin on AMP production in vivo. *Edin* RNAi and overexpression flies (*edin*,*Rel^E20^)* were crossed with *C564-GAL4* flies or *w^1118^* flies as a control, their offspring was infected with *E. cloacae*, total RNAs were extracted at indicated time points and qRT-PCR for the indicated genes was performed. (A) Expression of *edin* is knocked down in *edin* RNAi flies crossed to *C564*-*GAL4* driver flies. (B) *Edin* overexpression flies express *edin* at a physiological level. *Edin* overexpression flies crossed with *C564-GAL4* have slightly higher levels of *edin* compared to flies crossed with *w^1118^*. For (A–B) the data are pooled from 2 independent experiments, and n = 8 for each sample at each time point. (C–H) The effect of *edin* RNAi and overexpression on the production of *Diptericin B* (C), *Cecropin A1* (D), *Attacin B* (E), *Attacin A* (F), *Attacin C* (G) and *Drosocin* (H). n = 4 for each sample at each time point. Error bars represent the standard deviation of each sample.

To test the role of Edin in Imd pathway regulation *in vivo*, we monitored the Imd pathway-mediated *AMP* gene expression levels with qRT-PCR in *edin* RNAi flies and in *edin* overexpression flies we created. The overexpression flies were created by microinjecting the *pUAST-edin* construct into *Rel^E20^* mutant embryos. To analyze Imd pathway activity, *edin* RNAi (VDRC #14289) and *UAS*-*edin,Rel^E20^* flies were crossed with the *C564*-*GAL4* driver that targets transgene expression to the fat body in addition to some other organs [22]. The Imd pathway was then activated in week-old offspring by septic injury with *E. cloacae*. Flies crossed with *w^1118^* flies were used as controls. As shown in Figure 5A, *in vivo* RNAi of *edin* using the *C564-GAL4* driver strongly suppresses *edin* expression in whole flies, indicating that the UAS-RNAi construct is effective. *UAS*-*edin,Rel^E20^* flies crossed with the *C564*-*GAL4* driver showed expression levels comparable to the *E. cloacae* infected control flies (Figure 5B).

In agreement with our *in vitro* results, *in vivo* RNAi of *edin* did not show any clear effect in the expression levels of the tested *AMP* genes (two left-most panels, Figure 5C–H). There is a trend towards a minor decrease at the 4 h time points of the tested *AMP*s, excluding *Drosocin* (Figure 5H), but the decrease was statistically significant only with *Cecropin A1* (Figure 5D) and *Attacin B* (Figure 5E). We next tested whether overexpression of *edin* affects the production of AMPs via the Imd pathway. We compared *AMP* expression after septic injury with *E. cloacae* between *UAS*-*edin* flies crossed with *C564*-*GAL4* and *UAS*-*edin* flies crossed with *w^1118^* flies. We observed moderate increase only in *Drosocin* expression at the 8 h time point (68% increase for p<0.05) (Figure 5H). Noteworthy, *edin* expression did not activate *AMP* gene expression without a microbial challenge (see the 0 h time point in the rightmost panel in Figure 5C–H). This is in line with the results in S2 cells and rules out the possibility that Edin would function as a cytokine mediating immune response from the site of induction to other tissues (for example from hemocytes to the fat body). Based on these results, we conclude that Edin has no important role in the regulation of the Imd pathway activity either *in vitro* or *in vivo.*

### Edin has no potent antimicrobial properties in vitro or in vivo

**Figure 6:**
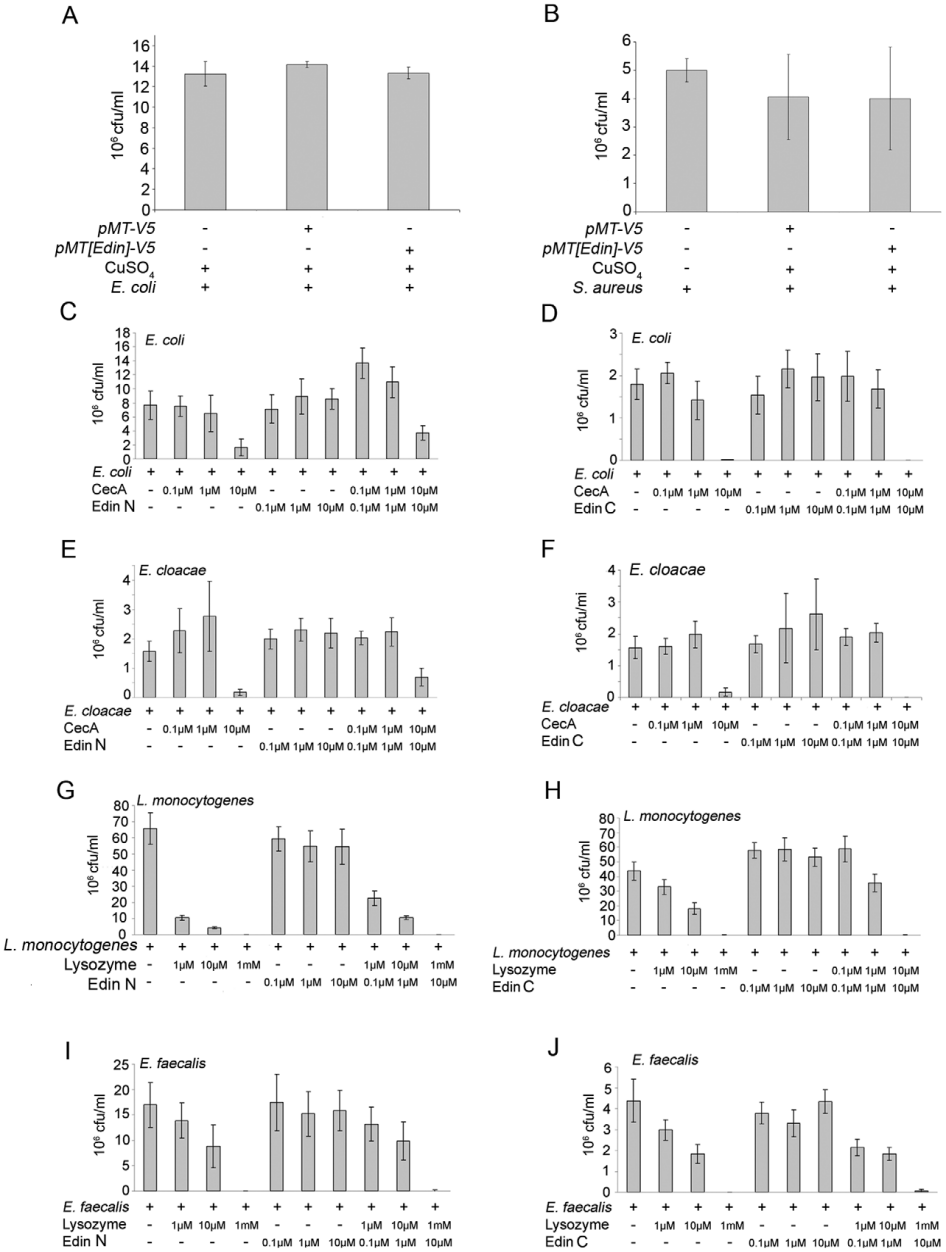
Edin has no broad antimicrobial properties against Gram positive or Gram negative bacteria in vitro. (A–B) Edin does no limit the growth of *E. coli* or *S. aureus* in S2 cell culture medium. S2 cells were transfected with a copper-inducible *pMT-edin-V5* or an empty *pMT* vector, and the abilities of *E. coli* and *S. aureus* to proliferate in these mediums were analyzed. (C–G) Synthetic forms of Edin do not limit the growth of *E. coli* (C), *E. cloacae* (D), *L. monocytogenes* (E), *E. faecalis* (F) or *S. aureus* (G). Both N-terminal and C-terminal forms of Edin were tested. Bacteria were cultured to an OD_600 nm_ of 0.33, incubated with synthetic Edin and the ability of the bacteria to grow was analyzed. Cecropin A and Lysozyme were used as positive controls for Gram-negative and Gram-positive bacteria, respectively. Left column, N-terminal Edin; right column, C-terminal Edin.

The kinetics of *edin* expression closely resembles those of known AMP genes, which led us to examine whether Edin has antimicrobial properties *in vitro* or *in vivo*. To study this, we first analyzed whether Edin was able to limit bacterial growth *in vitro*. We overexpressed *edin* in S2 cells, collected the cell culture medium and incubated the medium either with *E. coli* or *S. aureus*. Medium from S2 cells transfected with an empty vector was used as a control. As shown in Figure 6A and 6B, *E* *. coli* and *S. aureus* grew equally well in control medium and in medium containing Edin.

To further investigate the antimicrobial properties of Edin *in vitro*, we designed synthetic peptides containing the amino acids 22–45 (Edin C-terminal form) or 50–115 (Edin N-terminal form. The peptides were tested for their ability to reduce bacterial growth *in vitro*. Cecropin A and Lysozyme were used as positive controls for Gram-negative and Gram-positive bacteria, respectively. The peptides were incubated with *E. coli* (Figure 6C–D), *E. cloacae* (Figure 6E–F), *L. monocytogenes* (Figure 6G–H) or *E. faecalis* (Fig. 6I–J) and colony forming units were determined. As shown in Figure 6C–J, Cecropin A and Lysozyme at their highest concentrations almost abolished the growth of the tested microbes whereas neither the synthetic C-terminal or N-terminal form of Edin was able to affect the growth of the bacteria. Moreover, no synergistic effects were observed when Edin was incubated together with either Cecropin A or Lysozyme (three rightmost columns in Figure 6 panels C–J).

**Figure 7:**
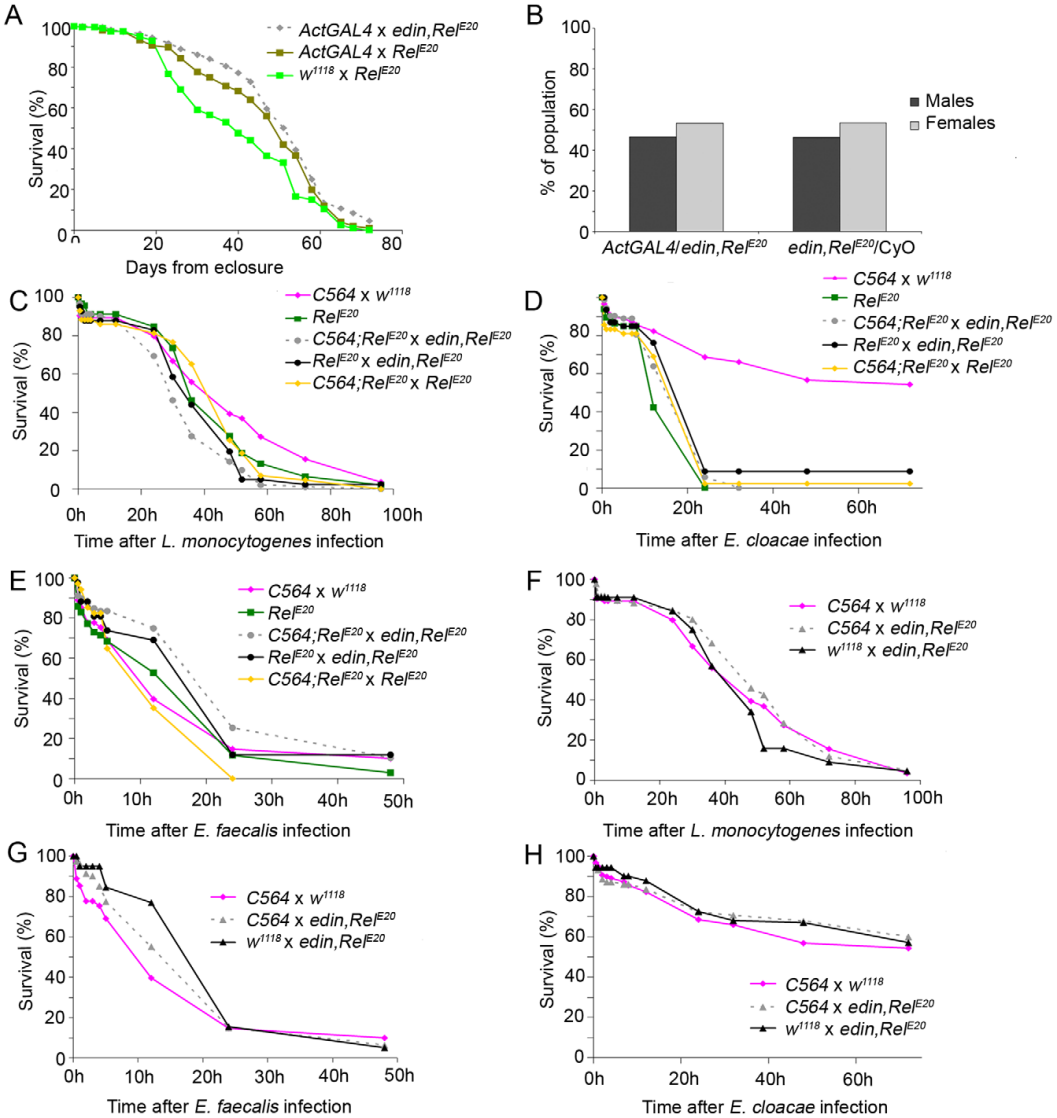
Overexpressing *Edin* has no effect on fly survival after Gram-positive or Gram-negative bacterial challenge in vivo. (A) Overexpressing *edin* does not negatively affect lifespan. *UAS*-*edin* overexpression flies were crossed with *Actin5C-GAL4* driver lines and the lifespan of their offspring was followed. *w^1118^* crossed with *Rel^E20^* mutants and *Act5C-GAL4* crossed with *Rel^E20^* were used as controls. The data represent one experiment, n = 100 for each cross. (B) Survival is not negatively affected in *UAS*-*edin* overexpressing flies. Equal amounts of *edin,Rel^E20^/Act5C-GAL4* and *edin,Rel^E20^/CyO* genotypes were obtained from the crosses. (C–H) Flies were pricked with the indicated microbe and survival was followed. (C–E) Overexpressing *edin* in the *Rel^E20^* background does not protect the flies from *L. monocytogenes* (C), *E. cloacae* (D) or *E. faecalis* (E) infection. In C–E *Rel^E20^* crossed with *edin*,*Rel^E20^* and *C564*;*Rel^E20^* crossed with *Rel^E20^* were used as controls. (F–H) Overexpressing *edin* in a heterozygous *w^1118^* background does not protect the flies from *L. monocytogenes* (F), *E. cloacae* (G) or *E. faecalis* (H) infection. *Edin* overexpression flies were pricked with *E. faecalis*, *E. faecalis* or *L. monocytogenes. C564-GAL4* flies crossed with *w^1118^* and UAS-*edin,Rel^E20^* crossed with *w^1118^* flies were used as controls. Data are pooled from 2–3 experiments which showed similar trends, for each cross (D–J) n = 34–118.

To test the antimicrobial properties of Edin in a more physiological context, the effect of *edin* overexpression on the survival of flies after bacterial infections was analyzed. First, to test whether overexpressing *edin* affects survival or lifespan, the *UAS*-*edin*,*Rel^E20^* overexpression line was crossed with the *Act5C-GAL4*/CyO driver line and the lifespan of the offspring was monitored. As shown in Figure 7A, overexpression of *edin* did not affect the lifespan of the flies and was comparable to that of the control flies. Furthermore, *edin* expression did not compromise the development of flies since equal amounts of *UAS*-*edin,Rel^E20^/ActGAL4* and *UAS*-*edin,Rel^E20^/*CyO flies were obtained from the crosses (Figure 7B). Similar results were obtained when *edin* overexpression flies where crossed with either the *C564*-*GAL4* driver line or the ubiquitous *daughterless-GAL4* driver line (data not shown).

An earlier study has shown that the expression of a single AMP can restore antimicrobial activity in *Drosophila* [23]. To test whether the expression of *edin* is sufficient to enhance resistance against septic infection in adult flies, we expressed *edin* in a homozygous *Rel^E20^* mutant background using a *C564-GAL4;Rel^E20^* line. In the homozygous *Rel^E20^* background, AMP production via the Imd pathway is eliminated making the flies very sensitive to infections with Gram-negative bacteria [24]. To test whether Edin had antimicrobial properties against Gram-negative or Gram-positive bacteria *in vivo*, we infected the *UAS-edin,Rel^E20^* flies crossed with the *C564-GAL4;Rel^E20^* driver with the Gram-positive bacterium *L. monocytogenes* (Figure 7C), which has a DAP-type peptidoglycan, with the Gram-negative bacterium *E. cloacae* (Figure 7D), and with the Gram-positive bacterium *E. faecalis* (Figure 7E). In this homozygous *Rel^E20^* background, overexpression of *edin* did not affect the survival rate upon septic injury with any of these microbes. In addition, no rescue was observed after a septic *E. coli* infection (data not shown). According to the results, e*din* overexpression was not sufficient to rescue the flies from succumbing to bacterial infection (Figure 7C–E) indicating that Edin alone does not possess sufficient antimicrobial properties against Gram-negative or Gram-positive bacteria.

To test whether Edin has antimicrobial properties in the context of a normal functioning immune response in *Drosophila*, we overexpressed *edin* in a heterozygous *Rel^E20^* mutant background. *Edin* overexpression flies crossed with *C564-GAL4* were infected with *L. monocytogenes* (Figure 7F), *E. cloacae* (Figure 7G) and *E. faecalis* (Figure 7H) and monitored for survival. As shown in Figure 7F–H, overexpressing *edin* did not protect the flies from the bacterial infection. Together these results indicate that Edin has no antimicrobial properties against either Gram negative or Gram positive bacteria *in vitro* or *in vivo*. These results argue that Edin has another immune response modulating function.

### Edin is required for normal resistance against bacteria

**Figure 8:**
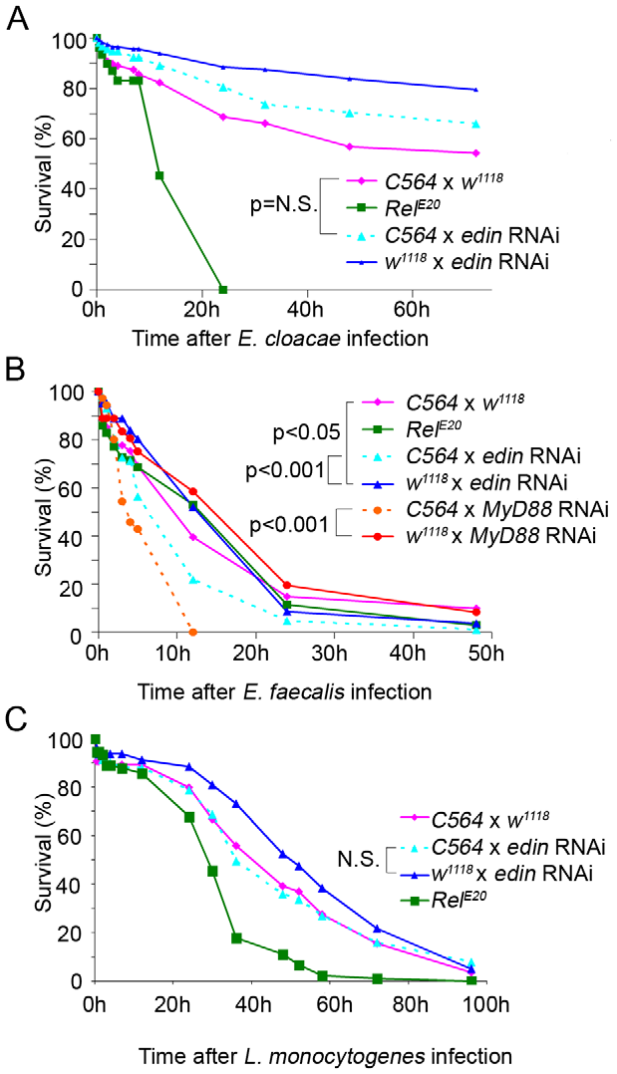
Edin RNAi impairs survival *in vivo* after *E. faecalis* infection. A–C. Healthy adult flies were pricked with a needle dipped either into a culture of *E. cloacae*, *E. faecalis* or *L. monocytogenes* and the survival of the flies was monitored. *Rel^E20^* mutants and/or *MyD88* RNAi flies were used as positive controls. (A) Effect of *edin* RNAi after *E. cloacae* infection. Data are pooled from 3 independent experiments which showed similar trends, n = 112–117 for each cross. (B) *Edin* RNAi flies crossed with the *C564-GAL4* driver are more susceptible to *E. faecalis* infection than uninduced *edin* RNAi flies crossed with *w^1118^*. Data are pooled from 2 independent experiments which showed similar trends n = 81–87 for each cross. For *MyD88* RNAi crossed to *w^1118^* and *C564*, data represents one experiment and n = 35 for both crosses. (C) *Edin* RNAi does not have a significant effect on fly survival against *L. monocytogenes* challenge. Data are pooled from 3 independent experiments which showed similar trends, n = 78–90 for each cross.

Next, we investigated whether Edin is required for normal resistance against septic infection. To this end *edin* RNAi flies were crossed with the *C564*-*GAL4* driver or *w^1118^* flies as a control, and the one-week-old offspring were infected with *E. cloacae*, *E faecalis* or *L. monocytogenes*. *Rel^E20^* mutant flies were used as a positive control in the *E. cloacae* and *L. monocytogenes* infection model, and *UAS*-*MyD88* RNAi flies crossed with the *C564*-*GAL4* driver as a positive control in the *E. faecalis* infection model. When infected with the Gram-negative bacterium *E. cloacae*, *Rel^E20^* mutant flies succumbed to the infection within 24 h. *Edin* RNAi flies crossed with *C564-GAL4* flies showed a mild decrease in survival after *E. cloacae* infection compared to *edin* RNAi flies crossed with *w^1118^* (Figure 8A) but this it not significant because the *C564-GAL4* driver flies crossed to *w^1118^* are more susceptible to the infection. However, a decrease in survival was observed in *edin* RNAi flies infected with the Gram-positive bacterium *E. faecalis* (Figure 8B). However, no statistically significant difference in survival was seen after an *L. monocytogenes* infection (Figure 8C), although a similar trend in survival could be observed, which is in line with the results by Gordon et al. [15]. These results imply that the expression of *edin* might be required for normal resistance against some bacterial infections.

## Discussion

In *Drosophila*, the expression of many genes is induced in response to microbial infection. In this study, we examined the role of the infection-inducible gene *edin* in the immune response of *Drosophila melanogaster* both *in vitro* and *in vivo*. We show that *edin* is highly induced in S2 cells by *E. coli* and its expression is dependent on the NF-κB transcription factor Relish both *in vitro* and *in vivo*. In line with the results of Verleyen and coworkers [16], we observe that Edin has a functional signal sequence leading to its cleavage and secretion from S2 cells. Despite the fact that *edin* is highly induced upon infection and that its expression pattern resembles that of known AMPs, we were not able to observe any antimicrobial properties *in vitro* or *in vivo*. Nor were we able to see any bacterial binding or opsonization when these properties of Edin were studied. *Edin* expression also was dispensable for AMP expression via the Imd pathway both *in vitro* and *in vivo*. However, interestingly *edin* RNAi flies showed decreased survival after bacterial infection with *E. faecalis*.

Traditionally, most studies on *Drosophila* AMPs have been successfully carried out *in vitro*. However, *Drosophila* is also a powerful model system for studying the activity of antimicrobial peptides *in vivo*, since it is easy to produce immunocompromised mutant fly lines, which are viable and fertile. Earlier studies have shown that *Drosophila* mutants of the Toll and Imd pathway, that have impaired production of AMPs via these signaling pathways, are highly susceptible to microbial infections [2], [4], [24] and even a single bacterial cell can be enough to kill a mutant fly [24]. The antimicrobial properties and the microbial specificity of a gene product can be studied by overexpressing the gene of interest in the mutant background of choice. It has been reported that the overexpression of a single antimicrobial peptide in Toll and Imd pathway double mutant flies can restore the resistance to a microbial infection to a level comparable to that of wild-type flies [23]. In our current study, we were not able to demonstrate a broad antimicrobial role for Edin *in vitro* or *in vivo*. *In vitro*, we observed no effect on the colony forming of bacterial cells when Edin was produced in S2 cells or when synthetic peptides were used.

*In vivo*, the effect of *edin* overexpression on the resistance against microbial infection was analyzed both in a homozygous *Rel^E20^* mutant background and in a heterozygous background. *Rel^E20^* mutants were selected since they are highly sensitive to Gram-negative bacterial infections. However, no increase in survival after septic injury could be observed in either one of these backgrounds. Therefore it is likely that Edin does not have an antimicrobial role in *Drosophila* although it is highly expressed upon bacterial infection. However, it is also possible that Edin is effective only against a specific microbe which we did not test in our current study. The *in vivo* analysis of antimicrobial properties of a certain peptide is further complicated by the production of a large battery of AMPs that can be partially redundant in their specificities. For instance, Edin alone might not be sufficient to fight against microbial infections, but it may require the presence of another AMP(s), or other immune effector molecules, for full activity.

Previously, Gordon and coworkers [15] have reported that high expression levels of *edin* are detrimental to fly survival and lifespan. We carried out lifespan experiments with our *edin* overexpression fly line and analyzed the proportions of the eclosed progeny. In contrast to Gordon et al., we did not observe a negative effect of *edin* overexpression on fly survival or lifespan. This difference in results could be due to different expression levels of *edin* or different genetic backgrounds of the flies used in these studies. According to our results, the *edin* overexpression fly line used in this study shows expression levels comparable to expression levels upon septic infection (Figure 5B). Furthermore, Gordon et al. [15] reported that Edin is required for resistance against *Listeria monocytogenes* infections. *L. monocytogenes* is a DAP-type peptidoglycan containing intracellular bacterium which can infect both mammals and *Drosophila* [25], [26]. Gordon et al. [15] report a significant decrease in survival after *L. monocytogenes* infection with two independent *edin* RNAi lines indicating that the normal *edin* expression is required for an efficient host response against the pathogen. In our current study, we did not observe a statistically significant reduction in the survival of *edin* RNAi flies after *L. monocytogenes* infection. However, the trend in the survival curve of *edin* RNAi flies is similar to that reported by Gordon et al. Since *Listeria* is an intracellular pathogen, Edin might also have an intracellular function although it is processed and secreted from the cell (Figure 1B). The processed form of Edin is also observed inside the cells (Figure 1B) which would support this hypothesis. However, further studies on the mechanisms involved in resistance against *Listeria* are required to elucidate the role of Edin in the infection.

We also analyzed the role of Edin as a modulator of innate immune signaling cascades. Nevertheless, our experiments indicate that Edin no strong effect on Imd pathway activity either *in vitro* or *in vivo*.

We conclude that the expression of *edin* is Relish-dependent both *in vitro* and *in vivo* but further studies are required to elucidate the exact role of Edin in the immune response in *Drosophila*. Also the mechanisms and signaling pathways involved in the *Listeria monocytogenes* infection remain to be studied.

## Materials and Methods

### Oligonucleotide microarrays

Oligonucleotide microarray expression data of S2 cells was collected from [14].

### Microbial culture

*Listeria monocytogenes* (strain 10403S), *Enterococcus faecalis*, *Staphylococcus aureus* and *Staphylococcus epidermidis* were cultured in BHI. *Enterobacter cloacae* (strain β12) and *Micrococcus luteus* were cultured in LB supplemented with either 15 ng/ml of nalidixic acid (Sigma-Aldrich, St. Louis, Missouri, USA) or 100 µg/ml of streptomycin (Sigma-Aldrich), respectively. *Serratia marcescens* (strain Db11) and *Escherichia coli* were cultured in LB supplemented with 100 µg/ml of ampicillin. The baker's yeast *Saccharomyces cerevisiae* (AH109) was grown overnight in YPDA medium (Gibco/Life Technologies, Carlsbad, CA, USA) supplemented with 15 µg/ml of kanamycin at +30°C with shaking.

### Semi-quantitative and quantitative RT-PCR

Semi-quantitative RT-PCR reactions for *edin*, *Attacin A and Act5C* were performed using Super-Script™ II One-Step RT-PCR with Platinum Taq kit (Invitrogen/Life Technologies, Carlsbad, CA, USA). The following primers were used: *Edin*: 5′-GTTCTCCAACAAGTGCGG-3′ (forward), and 5′- CAGAAATGCCAGGTGCCC-3′ (reverse); *Attacin A*: 5′-TTTGGCCTACAACAATGCTG-3′ (forward), and 5′-GCTTCTGGTTGGCAAACG-3′ (reverse); *Act5C:* 5′-CGAAGAAGTTGCTGCTCTGG-3′ (forward), and 5′-AGAACGATACCGGTGGTACG-3′ (reverse).

Quantitative RT-PCR was carried out using the QuantiTect SYBR Green RT-PCR kit (Qiagen) and an ABI7000 (Applied Biosystems) instrument according to the manufacturer's instructions. Results were analyzed with the ABI 7000 System SDS software version 1.2.3. The following primers were used: *AttB*, 5′-CAGTTCCCACAACAGGACC-3′ (forward) and 5′-CTCCTGCTGGAAGACATCC-3′ (reverse); *Drosocin*, 5′-TTCCTGCTGCTTGCTTGCG-3′ (forward) and 5′-TGGCAGCTTGAGTCAGGTG-3′ (reverse); *AttA*, 5′-GCATCCTAATCGTGGCC-3′ (forward) and 5′-GCTTCTGGTTGGCAAACG-3′ (reverse); *AttC*, 5′-CATCGTTGGCGTACTTGGC-3′ (forward) and 5′-TTGCTGGAAGCTATCCCGC-3′ (reverse); *CecA1*, 5′-CGTCGCTCTCATTCTGGC-3′ (forward) and 5′-GTTGCGGCGACATTGGC-3′ (reverse); *DptB*, 5′-GACTGGCTTGTGCCTTC-3′, and 5′-CCTGAAGGTATACACTCC-3′ (reverse); and *Edin*, 5′-CTCGTGTCCTGCTGTCTG-3′ (forward), and 5′-GCCTTCGTAGTTGTTCCG-3′(reverse).

### S2 cell culture and transfections

*Drosophila* hemocyte-like S2 cells [27] (obtained from Invitrogen/Life Technologies) were maintained in Schneider's Insect Cell Culture Medium (Sigma-Aldrich, St. Louis, Missouri, USA) supplemented with 10% FBS, 100 U/ml Penicillin and 100 µg/ml Streptomycin (Sigma-Aldrich) at +25°C. The cells were transfected using the Fugene® transfection reagent (Roche Applied Science, Penzberg, Germany) according to the manufacturer's instructions.

### Cloning and constructs

*Edin* was cloned into the pMT/V5-HisA (Invitrogen/Life Technologies) and pUAST [28] vectors using S2 cell cDNA as a template. The primers used were 5′-CAGAATTCATGTTCTCCAACAAGTGC-3′ and 5′-CAGGTACCTCAGAAATGCCAGGTGCC-3′ for pUAST, and 5′-CAGCGGCCGCATGTTCTCCAACAAGTGC-3′ and 5′-CACTCGAGGAAATGCCAGGTGCCCCG-3′ for pMT/V5-His.

### Western blotting

S2 cells were transfected with 0.5 µg of pMT[*edin*]-V5. Cells were harvested, pelleted and lyzed 24 h after addition of CuSO_4_. 25 µg of cell lysate and supernatant were electrophoresed in NuPAGE 12% Bis-Tris gel (Invitrogen Life Technologies), blotted on a nitrocellulose membrane, and detected by Western blotting using mouse anti-V5 primary Ab (Invitrogen/Life Technologies) and goat anti-mouse Ab HRP conjugates (Molecular Probes) together with ECL Plus Western blotting detection system (GE Healthcare Life Sciences, Uppsala, Sweden).

### Synthetic peptides

Two forms of synthetic Edin were ordered from Peptide 2.0. (Chantilly, VA, USA). Amino acid sequences: N-terminal form, SYRQ PYPEEF QTSPE QLLQ VAPLV; C-terminal form, SPEGG SVVVT ASKDNQ VGREAS VQYNHN LYSSG DGRGS IDAYA QASRN FDYNR NNYEG GIRGT WHF. The peptides were dissolved in H_2_O according to the manufacturer's instructions.

### Colony forming unit assay

Edin-V5 expressed in S2 cells: S2 cells in 48-well plates in an antibiotic-free medium were transfected with 0.5 µg of pMT-*edin*-V5 plasmid or an empty plasmid. Expression of the plasmid was induced 48 h later by adding CuSO_4_ to a final concentration of 300 µM. 100 µl of overnight grown bacterial suspension (OD_600 nm_ = 0.33, ∼1*10^6^ bacteria/ml) was centrifuged and resuspended in 1 ml of Schneider medium supplemented with 10% FBS. 50 µl of *E. coli* and *S. aureus* suspension were added to the wells 24 h after CuSO_4_ and incubated for 2 h at +25°C. Serial dilutions of the bacterial suspensions were made in sterile water. 20 µl droplets of each dilution were pipetted on LB (*E. coli*) or BHI (*S. aureus*) agar plates, the plates incubated overnight at +37°C and the bacterial colonies counted.

Synthetic forms of Edin: An overnight grown bacterial suspension (∼1*10^6^ bacteria/ml) was centrifuged and washed as above and resuspended in 5% DMSO. 5 µl of *E. coli*, *E. cloacae*, *L. monocytogenes* and *E. faecalis* suspension were added on the 96-well plates containing synthetic Edin at concentrations of 10 µM, 1 µM and 100 nM. Suspensions were incubated for 2 h at +25°C, after which serial dilutions were made in sterile water. Dilutions were plated as above and the bacterial colonies counted. Lysozyme and Cecropin A (Sigma-Aldrich, St. Louis, Missouri, USA) were used as a positive control for Gram-positive and Gram-negative bacteria, respectively.

### Luciferase reporter assays and dsRNA treatments

Luciferase reporter assays to analyze the Imd, Toll and JAK/STAT pathways, and dsRNA treatments were carried out as described earlier [29], [30].

### *Drosophila* stocks

The *edin* RNAi line (stock #14289) was obtained from VDRC and the *C564-GAL4* flies were a kind gift from from Prof. Bruno Lemaitre (Global Health Institute, EPFL, Switzerland). *CG32185* transgenic flies were generated by microinjecting the *pUAST-edin* construct to the *Rel^E20^* background in the Umeå Fly and Worm Transgene Facility. The genotype of the *edin* overexpression fly line is w;+;*UAS-edin,Rel^E20^*.

### Lifespan experiments

*UAS*-*edin* flies were crossed with *C564-GAL4*, *Actin5C-GAL4/CyO* and *Daughterless-GAL4* driver flies. *Rel^E20^* crossed with driver flies were used as a control. The lifespan of the offspring of the crosses was monitored at +25°C. Flies were moved to vials containing 5 ml of fresh fly food twice a week and their survival was monitored. Males and females were kept in separate vials, 10 to 20 flies per vial.

### Infection experiments

Infections were carried out by pricking one week-old healthy flies with a thin tungsten needle dipped in a concentrated pellet of either *Escherichia coli*, *Enterobacter cloacae* (strain β12), *Enterococcus faecalis* or *Listeria monocytogenes* (strain 10403S) which were grown overnight on culture plates.

### RNA extraction from flies

Quadruplicates of five flies (2 females and 3 males) were snap frozen on dry ice 0 h, 1 h, 4 h or 8 h post-infection. Flies were homogenized in TRIsure reagent (Bioline, London, UK) and total RNAs were extracted according to the manufacturer's instructions.

### Statistical analysis

Statistical analyses of results were carried out using one-way ANOVA. For survival experiments, Log Rank analysis was carried out and p<0.05 was considered to be significant.

### Flow cytometry

The amount of cell-associated microbes was analyzed using flow cytometry as described earlier [20].

### Binding assay

The binding assay for Edin was carried out essentially as described earlier [20] with minor modifications. In brief, S2 cells were seeded onto 24-well plates and transfected with 0.5 µg of pMT-*edin*-V5 plasmid or an empty pMT-V5 plasmid. CuSO_4_ was added 48 h later to a final concentration of 500 µM. Cells were harvested the next day and the supernatant was collected. 1 ml of overnight grown microbial culture was centrifuged and the pellet was washed 5 times with 1× PBS. 500 µl of medium containing either pMT-*edin*-V5 or empty pMT-V5 was added in the tubes containing the microbial pellets or latex beads treated with 0.4 M N-(3-dimethylaminopropyl)-N′-ethylcarbodiimide (EDC) and 0.1 M N-hydroxysuccinimide (NHS) and coated with BSA (10 mg/ml in PBS, pH 7.4). The samples were incubated for 1 h in an end-to-end rotator at +4°C. Thereafter the samples were washed five times with 1 ml of 1× PBS and the pellet was suspended in 20 µl of PBS. To detach the bound Edin from the microbial cells, SDS-PAGE sample buffer was added and the suspension was boiled for 10 min. The samples were centrifuged and 30 µl of the supernatant was loaded on to a 12% NuPAGE BisTris gel, electrophoresed and the proteins were transferred to a nitrocellulose membrane as described above.
